# RMEL3, a novel BRAF^V600E^-associated long noncoding RNA, is required for MAPK and PI3K signaling in melanoma

**DOI:** 10.18632/oncotarget.9164

**Published:** 2016-05-04

**Authors:** Lucas Goedert, Cristiano G. Pereira, Jason Roszik, Jessica R. Plaça, Cibele Cardoso, Guo Chen, Wanleng Deng, Vashisht Gopal Yennu-Nanda, Wilson A. Silva, Michael A. Davies, Enilza M. Espreafico

**Affiliations:** ^1^ Department of Cell and Molecular Biology, Faculty of Medicine of Ribeirão Preto, University of São Paulo, Ribeirão Preto, São Paulo, Brazil; ^2^ National Institute of Science and Technology in Stem Cell and Cell Therapy and Center for Cell-Based Therapy, Ribeirão Preto, São Paulo, Brazil; ^3^ Department of Melanoma Medical Oncology, The University of Texas MD Anderson Cancer Center, Houston, Texas, United States of America; ^4^ Clinical Oncology, Stem Cell and Cell Therapy Program, Ribeirão Preto Medical School, Ribeirão Preto, São Paulo, Brazil; ^5^ Department of Genetics, Ribeirão Preto Medical School, and Center for Integrative System Biology (CISBi-NAP/USP), University of São Paulo, Ribeirão Preto, São Paulo, Brazil

**Keywords:** lncRNA, ncRNA, TCGA, ENSG00000250961, ENST00000506106.1

## Abstract

Previous work identified RMEL3 as a lncRNA with enriched expression in melanoma. Analysis of The Cancer Genome Atlas (TCGA) data confirmed RMEL3 enriched expression in melanoma and demonstrated its association with the presence of BRAF^V600E^. RMEL3 siRNA-mediated silencing markedly reduced (95%) colony formation in different BRAF^V600E^ melanoma cell lines. Multiple genes of the MAPK and PI3K pathways found to be correlated with RMEL3 in TCGA samples were experimentally confirmed. RMEL3 knockdown led to downregulation of activators or effectors of these pathways, including FGF2, FGF3, DUSP6, ITGB3 and GNG2. RMEL3 knockdown induces gain of protein levels of tumor suppressor PTEN and the G1/S cyclin-Cdk inhibitors p21 and p27, as well as a decrease of pAKT (T308), BRAF, pRB (S807, S811) and cyclin B1. Consistently, knockdown resulted in an accumulation of cells in G1 phase and subG0/G1 in an asynchronously growing population. Thus, TCGA data and functional experiments demonstrate that RMEL3 is required for MAPK and PI3K signaling, and its knockdown decrease BRAF^V600E^ melanoma cell survival and proliferation.

## INTRODUCTION

Melanoma is the most aggressive form of skin cancer. Targeted therapies against BRAF^V600^ mutations, which are present in ~50% of metastatic melanomas, achieve impressive initial clinical responses and benefit, but the development of acquired resistance to these agents is almost universal [1]. The identification of additional melanoma oncogenic mechanisms initiated by oncogenic BRAF will facilitate the development of more long-term effective therapeutic approaches.

Among different molecular candidates, there is growing data to support that long noncoding RNAs (lncRNAs) play a significant role in this disease [2]. A diversity of lncRNAs was described to promote cell proliferation, migration and metastasis in melanoma cells [3–5]. lncRNAs commonly exhibit context-dependent activity and cell type-specific expression [6], reinforcing their possible application for therapeutic targeting.

Previous work from our laboratory identified RMEL3 (ENSG00000250961) as a potential lncRNA with extremely enriched and specific expression in melanoma [7]. Analysis of melanoma cells also suggested a positive correlation between RMEL3 expression and the presence of the BRAF^V600E^ mutation [7].

In the present study, we have investigated RMEL3 interaction networks to elucidate its significance in this disease. This study supports that RMEL3 knockdown inhibits MAPK and PI3K pathways in melanoma.

## RESULTS

### RMEL3 expression is enriched in melanoma and varies across disease progression

**Figure 1:**
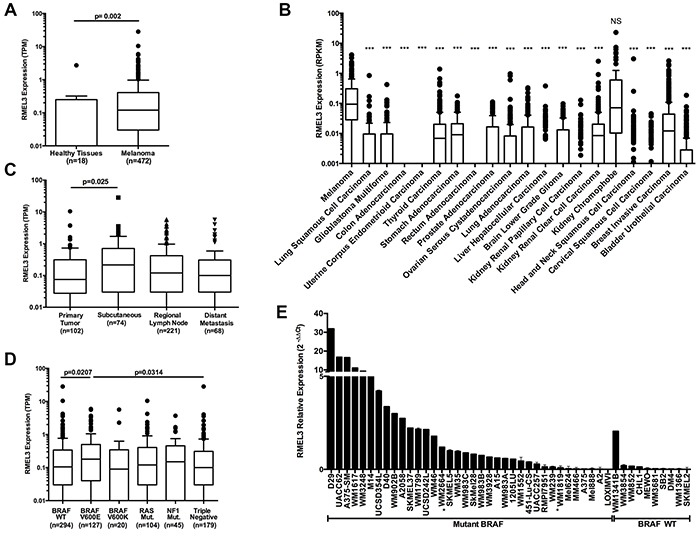
RMEL3 expression is enriched in melanoma, varies across tumor progression and is associated with BRAF^V600E^ **A.** TCGA melanoma, Illumina Body Map Project healthy tissues (adipose, adrenal, brain, breast, colon, heart, kidney, leukocyte, liver, lung, lymph node, ovary, prostate, skeletal muscle, testis and thyroid) and two melanocytes cell lines displaying RMEL3 expression*^#^. **B.** RMEL3 expression across different TCGA tumor types*^#^. **C.** TCGA patients classified by tumor tissue site and RMEL3 expression is displayed*^#^. **D.** TCGA patients classified according to gene somatic mutations: BRAF Wild Type (WT), BRAF^V600E^, BRAF^V600K^, RAS, NF1, and Triple negative for BRAF/RAS/NF1*^#^. **E.** Different melanoma cell lines harboring BRAF Wild Type (WT) or mutant BRAF (without asterisk are BRAF^V600E^; one asterisk are V600D) displaying RMEL3 relative expression measured with qRT-PCR and calculated with 2^−ΔΔCt^ method using TBP (TATA binding protein) as endogenous control. *Tukey's box-and-whisker plot. ^#^Mann-Whitney test assigned p-value between columns individual comparisons.

We analyzed the publicly available melanoma TCGA data to identify significant clinical and molecular associations of RMEL3 expression. Analysis of RNA expression data from 472 TCGA melanomas, 16 normal tissues (from Illumina Body Map Project) and 2 melanocytes (GSE38495) [8] confirmed significantly increased expression of RMEL3 in the tumors (Figure 1A). Also, RMEL3 expression is significantly greater in melanoma than a diversity of other tumors (Figure 1B). In clinical samples representing melanoma progression [primary tumors (n=102), subcutaneous tumors (regional cutaneous and in-transit metastasis, n=74), regional lymph node (n=221) and distant metastasis (n=68)], RMEL3 expression was increased in subcutaneous tumors compared to primary tumors (Figure 1C). RMEL3 expression was also significantly increased in melanomas with a BRAF^V600E^ mutation compared to those with a wild type BRAF or triple wild type for BRAF/RAS/NF1 [9] (Figure 1D), an association also observed in a panel of human melanoma cell lines (Figure 1E).

### RMEL3 knockdown decreases clonogenic capacity

**Figure 2:**
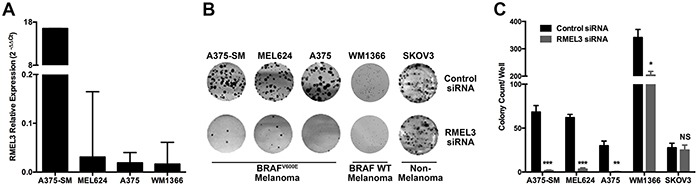
RMEL3 is required for cell clonogenic capacity **A.** RMEL3 relative expression measured with qRT-PCR and calculated with 2^−ΔΔCt^ method using TBP (TATA binding protein) as endogenous control in different melanoma cell lines. **B.** Clonogenic assay with RMEL3-silenced melanoma cell lines and with SKOV3 ovarian cancer cell line, which does not express RMEL3 **C.** Correspondent graph plotting of 3 biological replicates of clonogenic assays.

RMEL3 knockdown in BRAF^V600E^ melanoma cells, such as the A375-SM cell line, which has high RMEL3 expression (Figure 2A), markedly reduced colony formation (Figure 2B and 2C). BRAF^V600E^ RMEL3-low expressing cells (Figure 2A) are also affected (Figure 2B and 2C). RMEL3 knockdown in a BRAF wild type cell line also reduced colony count, however less dramatically. In contrast, SKOV3 ovarian cancer cell line, which has no RMEL3 expression, was not affected, demonstrating that the observed effects were not due to siRNA overall cytotoxicity or non-specific targeting (Figure 2B and 2C).

### RMEL3 expression alters melanoma cell expression profile

**Figure 3:**
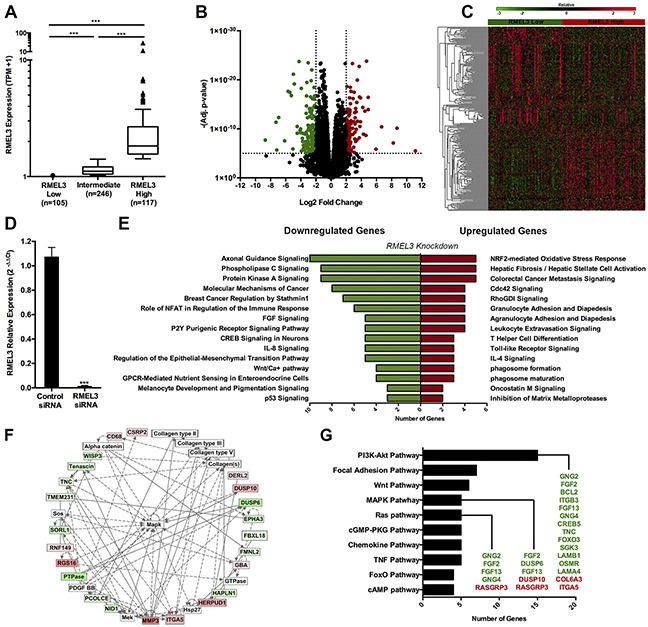
RMEL3 knockdown alters melanoma cell expression profile **A.** TCGA patients were classified into two groups of RMEL3 expressions. RMEL3 Low group (n=105), constituted of patients with RMEL3 expression below 25th percentile and RMEL3 High group (n=117), constituted of patients with RMEL3 expressions above the 75th percentile of the total RMEL3 expression set*. Tukey's box-and-whisker plot. **B.** Volcano plot and Heatmap **C.** displaying differentially expressed genes (log2 fold change <-2 or >2, adj. p-value <0.00001) between RMEL3 Low and High groups. **D.** A375-SM RMEL3 knockdown efficiency*. **E.** Canonical pathways enrichment of the validated genes by Ingenuity Pathway Analysis software. **F.** Cell-to-cell signaling and interaction network. Red squares are upregulated genes and green squares are downregulated after RMEL3 knockdown. White squares are connective molecules not differentially expressed. **G.** Pathways involvement of differentially expressed genes from TCGA analysis validated by RMEL3 knockdown. Green colored genes are downregulated after RMEL3 knockdown and red colored genes are upregulated. *Mann-Whitney test assigned p-value between columns individual comparisons.

To identify molecular features that are associated with RMEL3, two groups were separated according to RMEL3 expression levels from the total set of TCGA. RMEL3 Low group (n=105), constituted of patients with RMEL3 expression below 25th percentile and RMEL3 High group (n=117), constituted of patients with RMEL3 expression above the 75th percentile (Figure 3A). RNA-seq data was used to identify differentially expressed genes between the groups (log2 fold change <-2 or >2, adj. p-value <0.00001, n= 260 genes; Figure 3B and 3C; Supplementary Table S1).

Microarray analysis was performed following RMEL3 knockdown in the human melanoma cell line A375-SM (Figure 3D) to functionally validate genes regulated by RMEL3 (Supplementary Table S2). RMEL3-regulated genes identified by gene knockdown (log2 fold change <-0.5 or >0.5, p-value <0.01, n=2942; d-content>Supplementary Table S2) were compared to the melanoma TCGA data (log2 fold change <-0.5 or >0.5, adj. p-value <0.05, n=3445; Supplementary Table S3) to provide a high confidence list of RMEL3-correlated genes (Supplementary Table S4). A total of 177 genes positively differentially expressed in RMEL3 High tumors in the TCGA exhibited decreased expression following RMEL3 knockdown in the A375-SM cells. A set of 139 genes negatively differentially expressed in RMEL3 High group increased expression after RMEL3 knockdown. Pathway enrichment analysis of the validated genes implicates RMEL3 in protein kinase A signaling, molecular mechanisms of cancer, FGF signaling, regulation of epithelial-mesenchymal transition, inhibition of matrix metalloproteases, among others (Figure 3E, Supplementary Table S5). These pathways compose biological networks, as the example of cell-to-cell signaling and interaction, which has as central constituent the MAPK pathway (Figure 3F). Indeed, several validated genes are constituents of the PI3K-Akt, MAPK and Ras pathways (Figure 3G).

### RMEL3 influences melanoma critical proteins to promote cell cycle progression and survival

**Figure 4:**
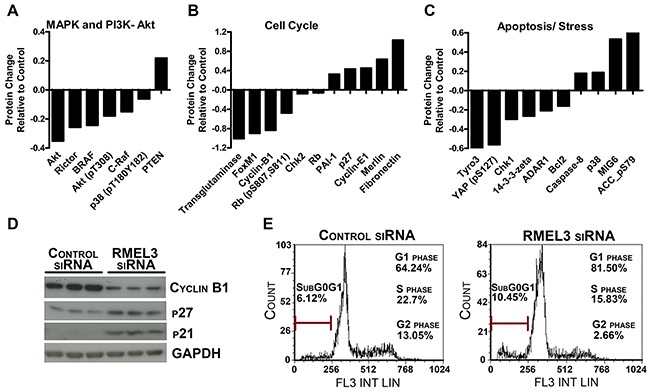
RMEL3 knockdown modulates important melanoma protein levels **A, B, C.** Protein relative concentration alteration assessed by RPPA in A375-SM RMEL3-silenced cells involved in the MAPK/ PI3K-Akt pathways, cell cycle and apoptosis. **D.** Western blot confirmation of important cell cycle regulatory proteins of A375-SM RMEL3-silenced cells. **E.** Cell cycle analysis of A375-SM RMEL3-silenced cells using propidium iodide as DNA stain.

The effects of RMEL3 knockdown on protein networks were assessed by Reverse Phase Protein Array (RPPA) analysis. RMEL3 knockdown in A375-SM melanoma cells altered a total of 91 proteins (p<0.05; Supplementary Table S6). We observed a decrease of the MAPK and PI3K-Akt components BRAF, Akt, pAkt (T308) and increased levels of PTEN (Figure 4A). RMEL3 knockdown reduced cell cycle regulators just as pRB (S807, S811) and Cyclin-B1, and increased p27 (Figure 4B). Pro-apoptotic proteins were increased, as the example of caspase-8 and p38, and anti-apoptotic proteins reduced, such as Bcl2 (Figure 4C). Consistent with the impact of RMEL3 knockdown in the clonogenic ability, we showed by western blot an increase of the p21 and p27 G1/S cyclin-Cdk inhibitors, and a decrease of cyclin B1 (Figure 4D). RMEL3 knockdown caused an accumulation of cells in the G1 cell cycle phase and an increase in cell death rates (subG0/G1) (Figure 4E). The observed results suggest that RMEL3 might work by suppressing mechanisms of G1/S checkpoint and apoptosis, thereby promoting cell cycle progression.

## DISCUSSION

In this work we confirmed previous findings of our group [7] that RMEL3 is highly restricted to melanoma (Figure 1A and 1B). A close correlation between RMEL3 and BRAF^V600E^ (Figure 1D and 1E) was observed and suggested that RMEL3 may be involved in cell proliferation and/or survival. Consistently RMEL3 knockdown induced a potent blockage over BRAF^V600E^ melanoma cell growth and survival (Figure 2A–2C).

RMEL3 validated genes are involved in pro-tumorigenic pathways, such as protein kinase A signaling, FGF signaling and regulation of epithelial-mesenchymal transition (Figure 3E). These results provide support to a possible involvement of RMEL3 in the in-transit metastasis process, evidenced by the increase of RMEL3 expression in the subcutaneous tumors (Figure 1C).

Importantly, several validated genes constitute components and transcription targets of the Ras-MAPK and PI3K-Akt pathways (Figure 3G), which are commonly active in melanoma [10]. Shared molecular signatures between RMEL3 knockdown and BRAF suppression also highlights the association of RMEL3 with BRAF. Common alterations are the upregulation of FOXD3 transcription factor [11]; WNT5A [12]; JUN, STAT3 [13]; fibronectin [14]; and other molecules involved in energy metabolism [15].

We also demonstrated, at the proteins levels, that RMEL3 knockdown decreased BRAF levels and increased the tumor suppressor PTEN concentration (Figure 4A), which commonly blocks BRAF^V600E^-induced malignancy [16, 17]. As expected, we detected reduced levels of pAkt (T308) and Akt (Figure 4A). This strong correlation with PI3K genes (Figure 3E and Figure 4A) would provide a powerful advantage to melanoma progression, since parallel activation of the PI3K may offset the negative feedback induced by ERK [18, 19] in BRAF^V600E^ cells [20].

RMEL3 knockdown also altered protein levels of cell cycle and apoptosis regulators. For instance, decreased protein levels of phosphorylated RB and cyclin-B1, as well as increased levels of the major effectors of G1 cell cycle arrest p21 and p27 were detected (Figure 4B and 4D). These results are consistent with the drastic reduction of clonogenic ability (Figure 2B–2C), G1 arrest and an increase in cell death rates (subG0/G1) (Figure 4E) caused by RMEL3 knockdown. Consistent with cyclin-B1 decrease, FOXM1 transcription factor that peaks in G2/M and activates cyclin-B1 expression [21] is also intensely downregulated (Figure 4B). On the other hand, the increase of cyclin-E1 after knockdown appears to be contradictory, however when targeted to the cytoplasm, cyclin-E1 promotes G1 arrest and senescence [22], which could be also reinforced by increased PAI-1 protein concentration (Figure 4B), a mediator of Ras senescence [23].

Interestingly, several changes tend to shift the balance towards apoptosis, including decrease of anti-apoptotic Bcl2 [24] mRNA expression (Supplementary Table S2) and protein levels (Figure 4C); an intense downregulation of transglutaminase protein that can inhibit bax-induced apoptosis [25]; increased pro-apoptotic p38 [26] and caspase-8 [27] protein levels; and decrease of YAP (pS127), which is phosphorylated by Akt to reduce p73-mediated induction of Bax expression [28]. Altogether, these protein changes may reflect inactivation of the critical MAPK and PI3K signaling pathways after RMEL3 knockdown.

In conclusion, this work provides strong evidence that RMEL3, initially implicated by its specificity to melanoma, can affect malignancy through MAPK and PI3K stimulation. Further characterization is ongoing to determine the mechanisms of RMEL3 functions and its potential value as a therapeutic target.

## MATERIALS AND METHODS

### TCGA data analysis

Skin Cutaneous Melanoma Clinical information and Exome (Level 2) data were directly downloaded from TCGA online platform (https://tcga-data.nci.nih.gov/tcga/) as December 2015. Analysis of TCGA RNA sequencing data was performed using the RSEM software [29]. We added RMEL3 exons positions (GRCh37): Exon1 (Chr5:56,690,887-56,691,005), Exon2 (Chr5:56,789,902-56,790,052), Exon3 (Chr5:56,792,607-56,792,744) and Exon4 (Chr5:56,828,763-56,829,251) to the input gene description database to be able to estimate the expression level of this gene. Raw RNA sequencing data from the TCGA skin cutaneous melanoma (SKCM) project were downloaded from the Cancer Genomics Hub (CGHub) [30]. MD Anderson MBatch batch effects portal (http://bioinformatics.mdanderson.org/main/TCGABatchEffects:Overview) was used to verify TCGA Batch effects and no RNAseq significant batch effects were found.

To compare expression levels, we used the transcripts per million (TPM) [31] results of RSEM. Melanocytes sequencing read data (GSE38495) were obtained from [8].

For differential expression analysis between RMEL3 High and Low expression groups it was used EdgeR package [32]. In all differential analysis, p-values were adjusted for False Discovery Rate (FDR) <0.05 as multiple hypothesis test correction method. Gene ontology was assessed with DAVID [33] and pathway enrichment with KEGG [34].

### Melanoma cell lines, RNA extraction and cDNA synthesis

Human melanoma cell lines were grown in RPMI media supplemented with 5% of fetal bovine serum (Life Technologies) and incubated at 37°C and 5% of CO2. For RMEL3 expression analysis, melanoma cells were seeded in 10 cm plates overnight, followed by cell lysis and RNA purification using the mirVana™ miRNA Isolation Kit (Ambion AM1560) according to manufacturer's instructions. Isolated RNA was treated with Dnase I (DNA-free kit, Ambion), quantified by spectrophotometer (NanoDrop® ND-1000 UV/Vis Spectrophotometer) and the amount of 1μg of the purified RNA was converted into cDNA using the High-Capacity cDNA Reverse Transcription kit (Applied Biosystems, CA).

### Expression analysis by qRT-PCR

Equal amounts of each cDNAs were used to verify RMEL3 expression by qRT-PCR with specific primers (5′>3′ sense ATGTGCTCCAAGAAAACCAGAG and antisense CTTTGTCACAGGAATACCCAAC) and SYBR Green PCR Power Mix 2x as detector agent (Applied Biosystems, CA) in a Mastercycler ep realplex real-time PCR system (Eppendorf). Cycle threshold (Ct) was converted to relative expression according to the 2^−ΔΔCT^ method, using TBP (TATA-box binding protein) as endogenous control.

### siRNA transfection

Cells seeded in 6-well plates a day before were transfected with different concentrations of siRNA using two transfection reagents, Dharmafect 1 (Dharmacon) or Xtremegene (Roche Applied Science). The parameters of transfection were set for each cell line and checked by Real Time PCR. Before leading to the subsequent experiments, the optimal condition was defined when the level of gene knockdown was higher than 90%. After appropriate treatments and incubations, cells were harvested for qPCR, western blotting, RPPA or used for clonogenicity analysis. The siRNA Stealth Universal Negative Control (Invitrogen) was used as a control, for RMEL3 a Stealth siRNA system (Invitrogen) was used. RMEL3 siRNA sequence: CCACUGCAGGGUUUCAGUCACAUGA.

### Gene expression profiling – microarray

Total RNA extracted as described above was subjected to whole-genome gene expression profiling using Sentrix HumanHT12 v4 beadchip arrays (Illumina). RNA amplification (TotalPrep RNA Amplification Kit, Life Technologies), array hybridization and data acquisition were performed at the University of Texas Health Science Center at Houston Microarray Core laboratory. The background was subtracted and arrays were quantile normalized. Differential expression analyses were performed among controls (cell lines transfected with siRNA Negative Control) and siRNA targeting RMEL3 gene using the GenomeStudio software (Illumina, Inc.). This data was uploaded to NCBI GEO platform as GSE72675.

### Reverse phase protein array

Cells were seeded in six well plates overnight and transfected in triplicates with control siRNA and RMEL3 siRNA. Protein lysates extracted from melanoma cells after 72 hours of transfection were quantified, normalized, denatured and submitted for RPPA analysis. Normalization and quality control were applied to RPPA data before further analyses (detailed description of the RPPA method and data normalization are available at http://www.mdanderson.org/education-and-research/resources-for-professionals/scientific-resources/core-facilities-and-services/functional-proteomics-rppa-core/index.html).

### Western blots

The same protein lysates used for RPPA were also used for Western Blot analysis. Briefly, 30μg of protein was fractionated by 10% SDS-PAGE and transferred to a nitrocellulose membrane. The membranes were incubated with blocking solution (5% Milk in TBS-T) for 1 hour at room temperature, and then blotted with relevant antibody (anti-p21; anti-p27 and anti-CCNB1 from Cell Signaling) diluted on a BSA 1% solution overnight at 4°C in the concentration of 1:1000. HRP-conjugated secondary antibody was detected by using the Enhanced Chemiluminescence Kit (GE Healthcare).

### Clonogenic assay

Cells transfected with control siRNA and RMEL3 siRNA were transferred into 6 well plates (330 cells/well) and cultured for 14 days at 37°C, 5% of CO2 atmosphere. Subsequently, the cells were fixed with 4% paraformaldehyde and stained with crystal violet 0.2% for colony visualization.

### Cell cycle

For cell cycle analysis, percentage of cells in each phase of the cycle was detected by propidium iodide staining. Cells kept for 72 hours after transfection with siRNAs in 24 wells plates were trypsinized, washed twice in PBS and fixed in ethanol overnight. Two hours before submitting the cells for analysis, the cells were washed in PBS twice following by incubation with propidium iodide solution (EMD Chemical) for 20 minutes at 37°C. Then, cells were analyzed on FACS Canto II flow cytometer (BD Biosciences) using BD FACSDiva software.

## SUPPLEMENTARY TABLES




