# Supplementary material for: RMEL3, a novel BRAFV600E-associated long noncoding RNA, is required for MAPK and PI3K signaling in melanoma
Source: Oncotarget. 2016 May 4;7(24):36711–8. doi: 10.18632/oncotarget.9164 (PMC5095033; doi:10.18632/oncotarget.9164)
Supplement: Supplementary file 1 [file oncotarget-07-36711-s001.pdf]

SUPPLEMENTARY TABLES

Supplementary Table S1: Differentially expressed genes between RMEL3 High X RMEL3 Low TCGA groups (adjusted p-value <0.00001 and log2 fold change -2< or >2).

See Supplementary File 1

Supplementary Table S2: Microarray of RMEL3 knockdown A375-SM melanoma cells (p-value <0.01 and log2 fold change -0.5< or >0.5).

See Supplementary File 2

Supplementary Table S3: Differentially expressed genes between RMEL3 High X RMEL3 Low TCGA groups (adjusted p-value <0.05 and log2 fold change -0.5< or >0.5).

See Supplementary File 3

Supplementary Table S4: Validated Genes (Differentially expressed genes between RMEL3 High and Low groups that were validated by microarray analysis of RMEL3-silenced cells).

See Supplementary File 4

Supplementary Table S5: Canonical Pathways Enrichment - Validated genes upregulated after RMEL3 knockdown.

See Supplementary File 5

Supplementary Table S6: RPPA of RMEL3 silenced A375-SM Melanoma cells.

See Supplementary File 6
